# Indoor mmWave Radar Ghost Suppression: Trajectory-Guided Spatiotemporal Point Cloud Learning

**DOI:** 10.3390/s25113377

**Published:** 2025-05-27

**Authors:** Ruizhi Liu, Zhenhang Qin, Xinghui Song, Lei Yang, Yue Lin, Hongtao Xu

**Affiliations:** 1 State Key Laboratory of Integrated Chips and Systems, Fudan University, Shanghai 201203, China; 20112020068@fudan.edu.cn (R.L.); 21307140085@m.fudan.edu.cn (Z.Q.); 22212020028@m.fudan.edu.cn (X.S.); 2 ICLegend Micro, Shanghai 201203, China; lei.yang@iclegend.com; 3 ICLegend Micro, Suzhou 215134, China; rick.lin@iclegend.com

**Keywords:** millimeter-wave radar, ghost suppression, multi-target tracking, point cloud segmentation, multipath

## Abstract

Millimeter-wave (mmWave) radar is increasingly used in smart environments for human detection due to its rich sensing capabilities and sensitivity to subtle movements. However, indoor multipath propagation causes severe ghost target issues, reducing radar reliability. To address this, we propose a trajectory-based ghost suppression method that integrates multi-target tracking with point cloud deep learning. Our approach consists of four key steps: (1) point cloud pre-segmentation, (2) inter-frame trajectory tracking, (3) trajectory feature aggregation, and (4) feature broadcasting, effectively combining spatiotemporal information with point-level features. Experiments on an indoor dataset demonstrate its superior performance compared to existing methods, achieving 93.5% accuracy and 98.2% AUROC. Ablation studies demonstrate the importance of each component, particularly the complementary benefits of pre-segmentation and trajectory processing.

## 1. Introduction

With the rapid development of smart homes and the Internet of Things (IoT), there is an increasing demand for efficient and accurate human detection. Smart applications such as presence-sensitive lighting and adaptive airflow systems—where lights respond to entry and exit, and fans adjust to follow or avoid a person—are creating demand for more precise and robust human detection technologies. Traditional human sensors have specific limitations. For example, passive infrared (PIR) sensors can only detect moving humans and are susceptible to thermal interference.

Millimeter-wave radar, with its high sensitivity to millimeter-scale displacements, can detect subtle movements such as breathing and even heartbeats [1,2], making it effective for detecting stationary human targets. Additionally, it provides rich information including range, angle, Doppler, and amplitude, enabling more complex applications such as fall detection [3,4]. However, due to multipath propagation and other effects, mmWave radar suffers from severe ghost (false detections) issues in indoor environments, reducing its reliability.

To mitigate ghost effects, researchers have proposed various approaches. Since ghosts are primarily caused by wall reflections, some methods rely on prior knowledge of wall geometry to distinguish real targets from multipath ghosts [5,6,7]. However, wall parameters are often unknown, prompting studies on wall estimation—for both single walls [8,9] and complex multi-wall environments [10,11,12,13].

Alternative methods eliminate the need for explicit wall parameter estimation. For instance, polarization-based approaches exploit changes in wave polarization upon reflection [14], though they require specialized antennas. Other techniques leverage linear patterns in range-Doppler spectra via the Hough transform [15]. Recent advances exploit differences between the direction of departure (DOD) and direction of arrival (DOA) for first-order multipath identification [16,17,18,19].

While physics-based methods offer strong interpretability, their reliance on simplified assumptions limits performance in complex multi-target, multi-wall scenarios. Consequently, data-driven approaches such as machine learning and deep learning have gained increasing attention. Some studies combine hand-crafted features with classifiers such as random forests (RFs), support vector machines (SVMs), or fully connected neural networks for ghost suppression [15,20,21,22,23]. Others take an image-based approach, using convolutional neural networks (CNNs) to process grid maps [24], range-Doppler maps [25], or DOD-DOA images [26]. Some works adopt point cloud-based methods, using networks such as PointNet and PointNet++ for point cloud segmentation [27,28,29,30].

To further enhance ghost suppression in complex environments, this paper proposes a method that integrates multi-target tracking with point-cloud-based deep learning. Although some existing works also incorporate tracking [5,18,21,31], they often rely on handcrafted features and utilize trajectory information through only simple heuristic rules. In contrast, our proposed method combines trajectory features with a point-cloud-based deep neural network, enabling automatic extraction of spatiotemporal features (combining spatial and temporal patterns across radar frames). Our key contributions include the following:

1. A robust tracking framework that associates detections across frames while maintaining trajectory consistency, even in challenging scenarios with closely spaced targets and ghosts.
2. An innovative trajectory feature aggregation network that combines PointNet-style point feature extraction with temporal CNN processing, enabling effective learning of spatiotemporal patterns.
3. A comprehensive system architecture that integrates preliminary segmentation, trajectory tracking, feature aggregation, and feature broadcasting to achieve state-of-the- art performance.

The experimental results demonstrate that our method achieves superior performance compared to existing point cloud segmentation approaches, with 93.5% accuracy and 98.2% AUROC on the test set. The ablation studies confirm the importance of each component, particularly showing that the combination of preliminary segmentation and trajectory-based processing captures complementary features that together improve the overall performance.

The remainder of this article is organized as follows. Section 2 introduces the basic theory of radar detection and ghost formation mechanisms. Section 3 details the proposed method. Section 4 presents the experimental results. Finally, Section 5 concludes this study.

## 2. Basic Theory of Radar and Ghosts

### 2.1. FMCW Radar

In frequency-modulated continuous-wave (FMCW) radar, the transmitted signal adopts a “chirp” waveform with a frequency that varies linearly over time. It is modeled as follows:

(1) $$s_{t}\left(t\right)=\exp\left(j2\pi \left(f_{0}t+\frac{B}{2T}t^{2}\right)\right),\;0\leq t\leq T,$$

where $f_{0}$ is the starting frequency, *B* is the sweep bandwidth, and *T* is the sweep time.

When the signal propagates to a target and reflects back, a round-trip time delay τ is introduced. After mixing the received signal with the transmitted signal and applying a low-pass filter, the resulting intermediate frequency (IF) signal is

(2) $$s\left(t\right)=\exp\left(j2\pi \left(\frac{B}{T}\tau t+f_{0}\tau \right)\right).$$

The IF signal is sampled by an analog-to-digital converter (ADC) with sampling interval $T_{s}$, producing $N_{s}=T/T_{s}$ samples per chirp. The radar transmits multiple chirps with period $T_{c}$ ($T_{c}>T$) over $N_{c}$ cycles, resulting in 2D data of the shape $\left(N_{c},N_{s}\right)$. For a uniform linear array with $N_{\mathrm{rx}}$ receivers spaced by $d_{\mathrm{rx}}$, the data forms a 3D cube of the shape $\left(N_{\mathrm{rx}},N_{c},N_{s}\right)$. The time delay τ satisfies

(3) $$\tau \left(n_{c},n_{\mathrm{rx}}\right)=\frac{2\left(R_{0}+vT_{c}n_{c}\right)+d_{\mathrm{rx}}\sin\theta \cdot n_{\mathrm{rx}}}{c},\;n_{c}\in \left[0,N_{c}\right),\;n_{\mathrm{rx}}\in \left[0,N_{\mathrm{rx}}\right).$$

where $R_{0}$ is the initial target distance, *v* is the radial velocity, θ is the azimuth angle, and *c* is the speed of light.

Substituting $t=T_{s}n_{t}$ and Equation (3) into Equation (2), we derive the following data cube expression:

(4) $$S\left(n_{t},n_{c},n_{\mathrm{rx}}\right)=\exp\left(j2\pi \left(f_{r}n_{t}+f_{v}n_{c}+f_{\theta }n_{\mathrm{rx}}+f_{0}\tau_{0}\right)\right),$$

(5) $$\mathrm{where}\;f_{r}=\frac{2B}{cN_{s}}R_{0},\;f_{v}=\frac{2T_{c}}{\lambda }v,\;f_{\theta }=\frac{d_{\mathrm{rx}}}{\lambda }\sin\theta ,\;\lambda =\frac{c}{f_{0}}.$$

The signal exhibits frequency characteristics along all three dimensions. By applying a fast Fourier transform (FFT) along each axis, the target parameters can be estimated.

**Figure 1:**
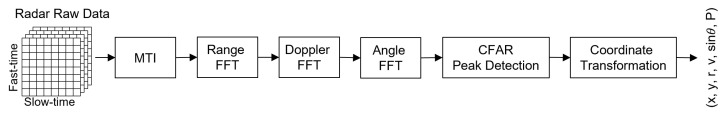
Flowchart of millimeter-wave radar signal processing.

The complete signal processing chain, as depicted in Figure 1, proceeds as follows: first, moving target indicator (MTI) processing is applied to the raw radar data to eliminate direct leakage between transmitters and receivers, as well as static clutter. Subsequently, a 3D-FFT is performed across the range, Doppler, and angular dimensions. Target extraction is then accomplished through 3D constant false alarm rate (CFAR) detection combined with peak identification. The detected frequency-domain peaks are converted into the following target parameters: range *r*, radial velocity *v*, sine of azimuth angle sinθ, and signal power *P*. Finally, the polar coordinates are transformed into Cartesian coordinates *x* and *y*. Consequently, each point in the point cloud contains six features: *x*, *y*, *r*, *v*, sinθ, and *P*.

### 2.2. Ghost Model

**Figure 2:**
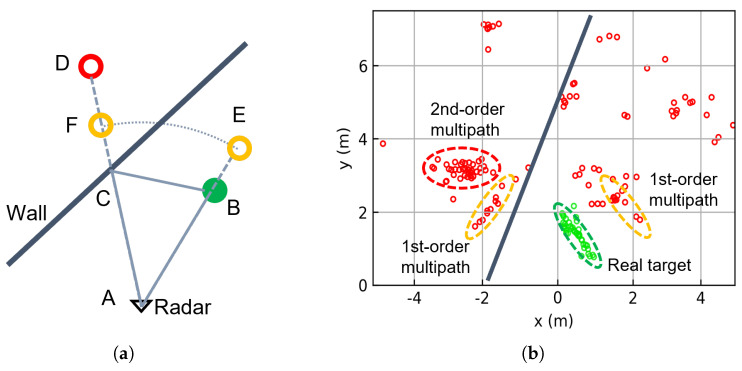
(**a**) Schematic of the geometry of multipath propagation. (**b**) Experimental multipath point cloud over a 2-s duration. Green circles indicate real targets, while red/yellow circles represent ghost targets.

Under ideal conditions, radar detects target positions by analyzing the reflected echoes, as illustrated by Path 1 (A→B→A) in Figure 2a. However, in real indoor environments, signal propagation becomes significantly more complex due to reflections from surrounding surfaces. These multipath effects are a major source of ghost targets. For example, Path 4 (A→C→B→C→A) produces second-order multipath ghost D, while Path 2 (A→C→B→A) and Path 3 (A→B→C→A) generate first-order multipath ghosts E and F, respectively.

We conducted an experimental validation of the multipath model using a 2T4R radar in real-world scenarios. Figure 2b clearly shows both first-order and second-order multipath phenomena. Second-order ghosts appear symmetrically positioned relative to the true target about the wall surface, while the first-order multipath produces two distinct clusters. These clusters maintain approximately equal distances from the radar, positioned between the true target and second-order ghost locations. One cluster shares the same direction as the true target, while the other aligns with the second-order ghost’s direction.

Compared to visible light, mmWave exhibit stronger specular reflection characteristics, resulting in more numerous ghost targets. This phenomenon stems from two key factors. First, according to the Fraunhofer criterion [32], surfaces become effectively smoother as the wavelength increases. In the mmWave band, only surfaces with millimeter-scale roughness, such as carpets, appear rough, while most other indoor surfaces behave like mirrors, creating numerous reflected ghost targets. Second, according to the Fresnel equations, reflection coefficients increase with higher relative permittivity. In general, materials exhibit lower permittivity at higher frequencies. Thus mmWave experiences higher reflectivity than optical frequencies, further contributing to ghost target formation.

The scenario becomes significantly more complex in environments with multiple targets and walls, making it increasingly difficult to distinguish real targets from ghosts. Physics-based modeling approaches struggle to simultaneously account for all of these interacting factors. Therefore, we employ a data-driven deep learning methodology to achieve more robust and reliable target discrimination.

## 3. Methods

**Figure 3:**
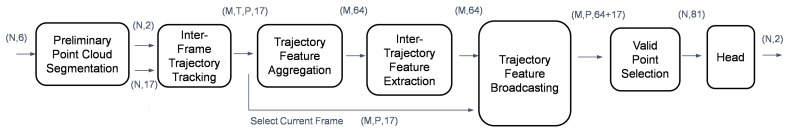
Overall pipeline of the proposed trajectory-based ghost target segmentation framework.

The ghost suppression problem can be equivalently formulated as a point cloud segmentation task, where each point is assigned a probability indicating whether it corresponds to a real target or a ghost. However, directly performing segmentation on each frame’s point cloud may lead to suboptimal performance. To address this, we propose a trajectory-based point cloud segmentation method. As illustrated in Figure 3, the overall framework consists of four stages: preliminary segmentation, inter-frame trajectory tracking, trajectory feature aggregation, and trajectory feature broadcasting.

### 3.1. Inter-Frame Trajectory Tracking

Due to fluctuations in target energy and other factors, it is challenging to identify ghosts using only single-frame point clouds. To improve the recognition rate, it is beneficial to accumulate multiple frames for joint analysis. As shown in Figure 2b, by aggregating over a period of time, the overall amount of information increases significantly. In theory, the more frames are accumulated, the better the recognition performance should be. However, increasing the number of frames also results in a larger number of points, which not only increases the computational complexity but also poses challenges for the scalability of the algorithm with respect to temporal accumulation.

To enhance the model’s ability to extract multi-frame information, we propose a tracking-based approach that maintains multiple trajectories and extracts features from them.

Each trajectory consists of four components: a trajectory ID, a first-in-first-out (FIFO) queue of associated historical points, a Kalman filter state, and a counter for consecutive unmatched frames. The most critical component is the FIFO queue, which has a depth of *T* and stores all points associated with the trajectory across the past *T* frames. Assuming that a maximum of *P* points can be associated per frame, the FIFO can hold up to T×P points.

Standard Kalman filtering [33] is used to estimate the current position and uncertainty of the trajectory, enabling more reliable data association. The Kalman state is defined using a 2D constant-velocity model:

(6) $$x=\left[\begin{array}{c} x\\ y\\ v_{x}\\ v_{y} \end{array}\right],\;P=\left[\begin{array}{cccc} \sigma_{x}^{2} & \sigma_{xy} & \sigma_{xv_{x}} & \sigma_{xv_{y}}\\ \sigma_{xy} & \sigma_{y}^{2} & \sigma_{yv_{x}} & \sigma_{yv_{y}}\\ \sigma_{xv_{x}} & \sigma_{yv_{x}} & \sigma_{v_{x}}^{2} & \sigma_{v_{x}v_{y}}\\ \sigma_{xv_{y}} & \sigma_{yv_{y}} & \sigma_{v_{x}v_{y}} & \sigma_{v_{y}}^{2} \end{array}\right].$$

**Figure 4:**
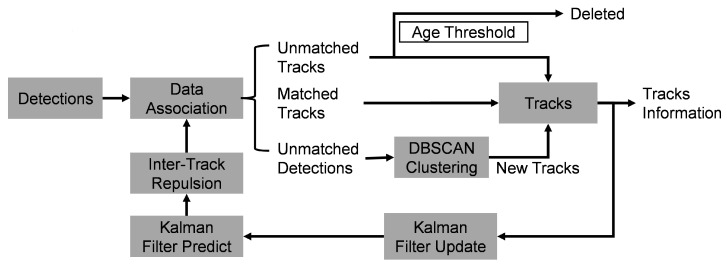
Inter-frame trajectory tracking pipeline.

As shown in Figure 4, the detailed process of multi-target tracking is as follows:

1. Assume that *N* targets are detected by radar in each frame, and each target has *C* features. These detections are associated with existing trajectories. During association, each detection searches for the closest trajectory in spatial distance. If the distance between them is less than the threshold $d_{\mathrm{th}}$, they are associated with each other.
2. Each detection can be associated with at most one trajectory, but each trajectory may be associated with multiple detections. These associated detections are added to the trajectory’s FIFO and used to update its Kalman filter state.
3. If a trajectory is not associated with any detection, a null value is added to the trajectory’s FIFO; if no detection is associated with the trajectory for $T_{\mathrm{del}}$ consecutive frames, the trajectory is deleted.
4. For detections not associated with any trajectory, DBSCAN clustering [34] is performed ($eps=d_{\mathrm{th}}$, MinPts=1). Each cluster creates a new trajectory; the points in the cluster are added to the trajectory’s FIFO and used to initialize the Kalman state. Setting MinPts=1 allows each unassociated detection to potentially form its own cluster, ensuring that no detection is discarded and every point can be assigned to a trajectory for subsequent processing.
5. After all detections have been associated, each trajectory performs a Kalman prediction step to update its state and uncertainty for the next frame.
6. Since one human body is often detected as multiple points, to avoid two trajectories tracking the same person, a repulsion mechanism is introduced. If the distance between two trajectories is less than $d_{\mathrm{th}}$, they are forcibly repelled by adjusting their positions to make the distance equal to $d_{\mathrm{th}}$. Here, the position refers to the position in the Kalman state. The repulsion is asymmetric, depending on the size of the Kalman state variance. A trajectory with higher uncertainty (i.e., a larger trace of the covariance matrix) is adjusted more, reflecting its lower reliability:
  (7) $$\Delta p_{i}=(d_{\mathrm{th}}-∥p_{i}-p_{j}∥)\cdot \frac{p_{i}-p_{j}}{∥p_{i}-p_{j}∥}\cdot \frac{\mathrm{tr}(P_{j})}{\mathrm{tr}\left(P_{i}\right)+\mathrm{tr}\left(P_{j}\right)}$$
  where $p_{i}={\left[x_{i},y_{i}\right]}^{T}$ is the position of track *i*, and $p_{j}$ is that of a nearby track *j*; tr(P) denotes the trace of the covariance matrix.
7. After the above steps, the tracking module completes all operations for the current frame. Finally, the contents of each trajectory’s FIFO are output. In this way, an input point cloud of the shape (N,C) is transformed into trajectory data of the shape (M,T,P,C), where *M* denotes the number of trajectories, *T* denotes the number of historical frames stored in each trajectory, *P* denotes the number of associated points per trajectory per frame, and *C* denotes the number of features per point.

**Figure 5:**
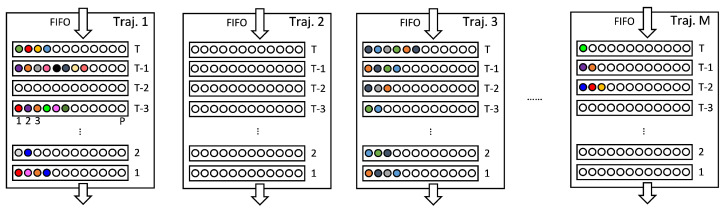
Structure of the trajectory FIFO. Colored circles: detections; empty circles: free slots.

The tracking parameters are set as follows: association threshold $d_{\mathrm{th}}=60$ cm, deletion delay $T_{\mathrm{del}}=9$, and trajectory buffer depth T=30. These key parameters are empirically selected; $d_{\mathrm{th}}$ and $T_{\mathrm{del}}$ are tuned based on visual inspection of stable tracking results on the dataset, while *T* balances temporal context with memory and computation requirements in the subsequent network. Other parameters are determined by the system characteristics; N=24 is based on the radar point cloud density, and M=32 (maximum number of trajectories) and P=12 (maximum number of points per trajectory per frame) are chosen to cover typical scene demands, with zero-padding applied when necessary. The data modality within the FIFO is illustrated in Figure 5.

After this process, all detections are assigned to a trajectory. This operation can be viewed as a form of clustering, where the clustering is implicitly performed in a temporally aware manner.

### 3.2. Preliminary Point Cloud Segmentation

Due to the presence of a considerable number of ghost targets in the point cloud, directly performing tracking on the raw data may lead to trajectory confusion. This issue is particularly prominent in narrow spaces such as corridors, where some ghost points may appear spatially close to real targets, causing the trajectory to be dragged by ghosts during the tracking process. To mitigate such effects, we apply frame-wise preliminary segmentation to the point cloud before tracking in order to suppress the interference caused by ghost targets to some extent.

The point cloud segmentation assigns each point a class probability, denoted as $P_{n}$ and $1-P_{n}$, representing the likelihoods of being a real target and a ghost, respectively, where n∈[0,N). This probability is later used in the Kalman measurement update to adjust the measurement noise covariance matrix $R_{n}$, which quantifies the uncertainty of point *n*’s position estimation.

**Figure 6:**
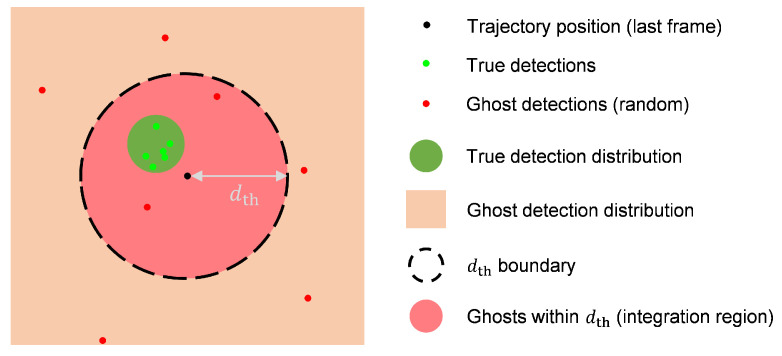
Geometric relationship of trajectories and detections with threshold $d_{\mathrm{th}}$.

As shown in Figure 6, when a ghost point falls within the $d_{\mathrm{th}}$ range of a trajectory, it will be associated with that trajectory. Assuming that ghost points are uniformly and randomly distributed, the equivalent measurement noise covariance of a ghost point, denoted as $R_{f}$, satisfies

(8) $$R_{f}=\left[\begin{array}{cc} \sigma_{x}^{2} & 0\\ 0 & \sigma_{y}^{2} \end{array}\right]$$

(9) $$\sigma_{y}^{2}=\sigma_{x}^{2}=E\left[{\left(x-\bar{x}\right)}^{2}\right]=E\left[{\tilde{x}}^{2}\right]=\int_{0}^{2\pi }\int_{0}^{d_{\mathrm{th}}}{\left(\tilde{r}\cos\tilde{\theta }\right)}^{2}\cdot \frac{1}{\pi d_{\mathrm{th}}^{2}}\;\tilde{r}\;d\tilde{r}\;d\tilde{\theta }=\frac{d_{\mathrm{th}}^{2}}{4}$$

where $\tilde{x}$ denotes the position offset from the trajectory center in the *x*-direction, and $\left(\tilde{r},\tilde{\theta }\right)$ are the corresponding polar coordinates centered at the trajectory. The integral assumes a uniform probability density $\frac{1}{\pi d_{\mathrm{th}}^{2}}$ over the circular region of radius $d_{\mathrm{th}}$.

Thus, we have $R_{f}=\frac{d_{\mathrm{th}}^{2}}{4}I_{2}$, where $I_{2}$ is the 2×2 identity matrix. For a point with real target probability $P_{n}$, the mixed measurement covariance is defined as

(10) $$R_{n}=P_{n}R_{t}+\left(1-P_{n}\right)\frac{d_{\mathrm{th}}^{2}}{4}I_{2}$$

where $R_{t}$ is the measurement noise covariance of real targets. In this work, we set $R_{t}=0.01\;I_{2}$, which is a small value. This linear mixing provides a soft transition between the low-noise model for real targets and the high-uncertainty model for ghosts, enabling more robust Kalman filtering under uncertainty. The use of adaptive measurement noise, combined with the trajectory repulsion mechanism, helps the tracker focus on high-confidence measurements and improves robustness in the presence of ghost targets.

In general, point cloud segmentation can be addressed using classical deep learning architectures such as PointNet [35]. The PointNet segmentation framework is defined as follows:

(11) $$f\left(x_{i},X\right)=g\left(x_{i},\max_{j\in [1,N]}h\left(x_{j}\right)\right)$$

where $x_{i}$ denotes the feature vector of the *i*-th point to be segmented, with i∈[1,N] and *N* being the total number of points. $f(x_{i},X)$ represents the segmentation result for point $x_{i}$, and $X=\{x_{1},x_{2},\ldots ,x_{N}\}$ is the entire point cloud. The functions g(·) and h(·) are nonlinear mappings implemented by multilayer perceptrons (MLPs).

In this work, we employ a deep-learning-based method for preliminary segmentation, namely, PairwiseNet, which was proposed in [30] and demonstrates stronger feature extraction capabilities than those of PointNet. Its formulation is given by

(12) $$f\left(x_{i},X\right)=g\left(\underset{j\in [1,N]}{\mathrm{agg}}\;h\left(x_{i},x_{j}-x_{i}\right)\right),$$

where agg denotes the aggregation operation, which can be implemented as either max pooling or average pooling.

**Figure 7:**
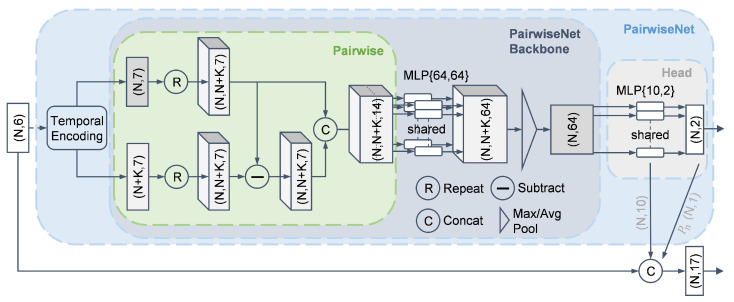
Architecture of PairwiseNet for preliminary point cloud segmentation.

As shown in Figure 7, PairwiseNet employs a “Pairwise” operation to establish point-wise correlations in the point cloud, subsequently aggregating features through shared-weight MLPs and pooling operations. Further implementation specifics can be found in [30].

The design rationale of PairwiseNet in [30] is that each ghost point typically originates from a real target (i.e., has a source); thus, pairwise operations are introduced to model such relationships. Considering the randomness and sparsity of radar point clouds, PairwiseNet in [30] adopts multi-frame accumulation and temporal encoding to further enhance segmentation performance. Although PairwiseNet is capable of leveraging multi-frame information, it is not well suited for processing long-term dynamic point clouds directly. This limitation stems from its pooling-based feature aggregation mechanism, which lacks the ability to effectively capture temporal dependencies and struggles to extract time-varying features.

This work retains the pairwise design rationale but differs in scope; while the authors of [30] apply it at the point level, we extend it to the trajectory level. Specifically, our method first extracts rich trajectory features and then applies PairwiseNet across trajectories (details are provided in the next section). The trajectory-based method proposed in this paper is specifically designed to enhance temporal feature extraction capabilities, thereby complementing the weaknesses of PairwiseNet in this regard.

In addition to being used for measurement covariance estimation to reduce tracking errors, the segmentation results from PairwiseNet are also employed for feature enhancement. As illustrated in Figure 7, the point-wise features from the head layer of PairwiseNet, along with the predicted probability $P_{n}$, are concatenated with the original point features. Consequently, the number of features per point increases from 6 to 17.

### 3.3. Trajectory Feature Aggregation

Based on the method described in Section 3.1, we obtain trajectory-aligned data with the shape (M,T,P,C), where C=17. This data modality is structurally complex; the *M*-dimension corresponds to different trajectories, exhibiting permutation invariance and sparsity; the *T*-dimension represents time, which contains local temporal correlations and a degree of translational invariance; the *P*-dimension corresponds to point cloud samples per frame, which is also unordered and sparse.

To extract meaningful representations from this structure, we propose a trajectory feature aggregation network that applies different aggregation strategies tailored to the characteristics of each dimension.

**Figure 8:**
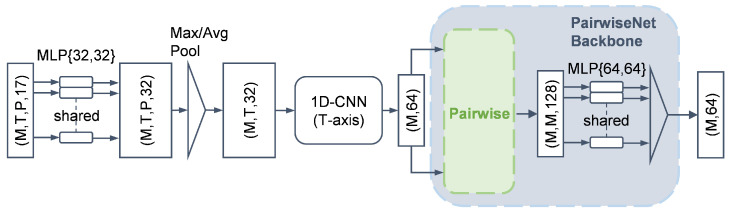
Trajectory feature aggregation pipeline.

As shown in Figure 8, we first apply feature aggregation along the point cloud dimension *P*. Inspired by PointNet, we adopt a shared-weight MLP followed by a pooling layer, which is well suited for handling unordered point cloud data. This step is crucial because the subsequent CNN requires structured inputs and cannot directly operate on sparse, unordered point sets. After pooling, the feature dimension is reduced to (M,T,32), where each trajectory at each time step is represented by a 32-dimensional feature vector.

Next, along the temporal dimension *T*, we apply a downsampling convolutional neural network (CNN) to exploit the translational invariance and extract local temporal features. The detailed architecture is listed in Table 1. To reduce the number of parameters and computational cost, each convolutional layer is implemented using depthwise-separable convolution (DSC) [36]. After passing through three DSC layers, the output is flattened into a feature vector. As a result, we obtain a feature tensor of shape (M,64), where each trajectory is encoded into a compact 64-dimensional representation.

### 3.4. Inter-Trajectory Feature Extraction

To incorporate the global context along the trajectory dimension *M*, we again utilize the PairwiseNet backbone. As illustrated in Figure 8, the input tensor of the shape (M,64) is pairwise-combined to form a tensor of the shape (M,M,128), representing all trajectory pairs. This pairwise structure is identical to that in Figure 7, consisting of two repetitions, one subtraction, and one concatenation operation. The resulting tensor is then passed through a shared-weight MLP followed by a pooling layer, resulting in a final output of the shape (M,64).

### 3.5. Trajectory Feature Broadcasting

Each detection is associated with a specific trajectory, and each trajectory may contain multiple detections. To utilize trajectory-level features for segmentation, we broadcast the feature vector of each trajectory to all of its associated detections.

In real-time processing scenarios, we are often only interested in the current frame. Therefore, we select the current time step from the temporal dimension *T*, resulting in detection data of the shape (M,P,17). At this stage, the historically informed trajectory features obtained in the previous section—summarizing each trajectory over *T* frames and shaped as (M,64)—are broadcast (or repeated) to match the shape (M,P,64) and concatenated with the current frame’s detection features to obtain data of the shape (M,P,81). In this way, each detection is augmented with the feature of its corresponding trajectory.

**Figure 9:**
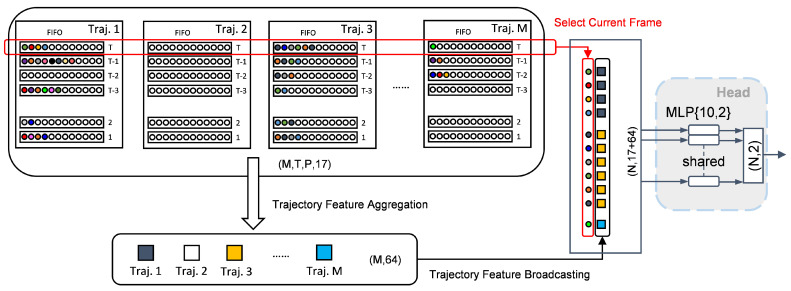
Trajectory-to-detection feature broadcasting.

Since the *M* and *P* dimensions may include zero-padded empty trajectories and empty points, we remove all invalid entries and retain only valid detections, resulting in a final feature tensor of the shape (N,81), where *N* corresponds to the original number of points in the frame. The effect of this broadcasting operation is illustrated in Figure 9.

Finally, these enriched point features are fed into a shared-weight MLP classification head to generate the segmentation output of the shape (N,2).

The mechanism of broadcasting aggregated trajectory features back to the individual points within that trajectory enables each point to incorporate not only its instantaneous features but also the spatiotemporal context derived from the entire trajectory. As a structured and efficient form of spatiotemporal information in multi-target systems, trajectories exhibit strong clustering capability for real targets. This context enhances segmentation performance beyond what instantaneous features or pre-segmentation alone can achieve.

## 4. Experiments and Evaluation

### 4.1. Dataset

The experiments in this paper were conducted on the indoor radar ghost dataset proposed in [30]. The dataset was collected using a 24 GHz 2T4R mmWave radar (ICLegend Micro, China) equipped with an eight-channel equivalent uniform linear array. It operates with a 250 MHz bandwidth, corresponding to a range resolution of 60 cm.

**Figure 10:**
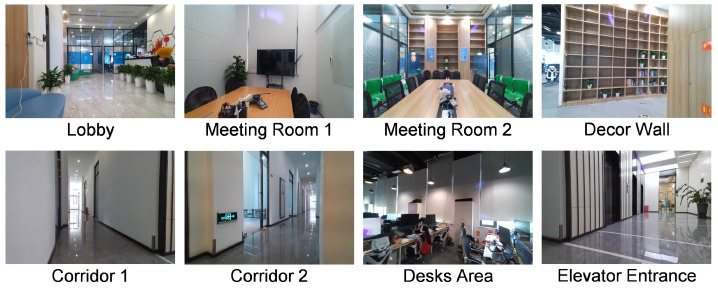
Representative scenes from the indoor radar ghost dataset.

The dataset contains 63 scenes and a total of 80,355 radar frames. The scenarios cover a variety of indoor environments, including halls, corridors, meeting rooms, and office areas, as illustrated in Figure 10. The radar platform is placed on the floor, tables, or cabinets, with mounting heights ranging from 40 cm to 150 cm to introduce diversity in installation locations. Each scene contains 0 to 5 people, who are allowed to move freely. Radar point clouds are annotated using an Azure Kinect DK (Microsoft Corporation, Redmond, WA, USA) depth camera. In total, the dataset contains 461,383 valid annotated points, among which 296,247 are real targets, and the remaining are ghost points.

### 4.2. Evaluation Metrics

For point cloud segmentation tasks, we adopt multiple evaluation metrics to assess model performance. The primary metric is point-wise classification accuracy, defined as the ratio of correctly classified points to the total number of points. Additionally, the precision, recall, and F1-score of the real target category are also used as metrics.

In practical applications, the costs associated with false positives and false negatives often differ significantly. This imbalance necessitates careful selection of thresholds to achieve an optimal trade-off between these two types of errors. For system performance evaluation, we use receiver operating characteristic (ROC) curve analysis, which visually illustrates the relationship between true positive detection rates and false alarm rates across different decision thresholds. The area under the ROC curve (AUROC) serves as a reliable indicator of overall performance. Additionally, average precision (AP) is also employed as a metric, which is the area under the precision–recall curve.

### 4.3. Experimental Setup

For the preliminary point cloud segmentation, we directly reuse the pretrained weights of PairwiseNet provided in [30], without retraining. Based on the segmentation results and the tracking method described in Section 3.1, we generate the trajectory data for each frame in the entire dataset, resulting in a tensor of the shape $\left(B_{\mathrm{all}},M,T,P,C\right)$, where $B_{\mathrm{all}}$ denotes the total number of frames. These data are used to train the trajectory-based point cloud segmentation network, which includes trajectory feature aggregation and broadcasting modules and is referred to as TrajNet in this paper.

The loss function used during training is the cross-entropy (CE) loss, defined as

(13) $$L_{CE}\left(y,\hat{y}\right)=-\frac{1}{N_{\mathrm{eff}}}\sum_{i=1}^{N_{\mathrm{eff}}}\left[y_{i}\log\left({\hat{y}}_{i}\right)+\left(1-y_{i}\right)\log\left(1-{\hat{y}}_{i}\right)\right]$$

where $y_{i}$ is the ground-truth label (0 or 1) of the *i*-th point, ${\hat{y}}_{i}$ is the predicted probability of being a real target, and $N_{\mathrm{eff}}$ is the number of valid points in the point cloud. Placeholder points (zero-padded entries) are excluded from the loss and all evaluation metrics.

We use the Adam optimizer during training, with exponential decay rates of 0.9 and 0.999 for the first- and second-order moment estimates, respectively. The learning rate is set to 0.003, and the batch size is 64.

The 63 scenes in the dataset are split into 45 for training, 11 for validation, and 7 for testing, which is consistent with the split in [30]. This ensures that the test set consists of entirely new scenes that are not seen during training. The training set is used for gradient descent updates, while the validation set is used for early stopping to prevent overfitting. The AUROC is calculated on the validation set after each epoch. If the AUROC score stops improving and starts to decline, training is terminated early, and the best-performing model parameters are saved. The maximum number of training epochs is set to 50.

### 4.4. Trajectory Visualization

**Figure 11:**
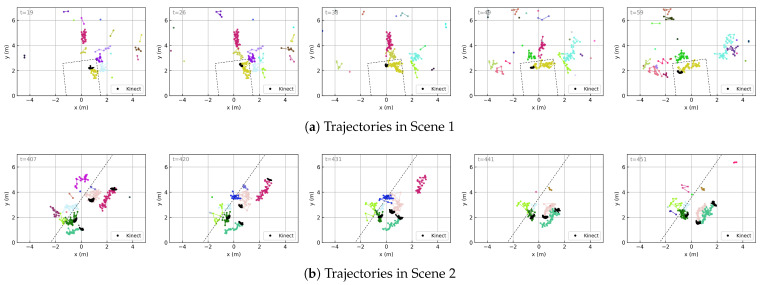
Trajectory evolution over time. The frame number is indicated in the top-left corner of each subfigure and increases from left to right. Dashed lines represent major reflective surfaces (i.e., walls). Curves of different colors represent different trajectories, and the points along each curve are the detections associated with that trajectory. Dense clusters of small black points indicate ground-truth human positions obtained through the Kinect, serving as a reference for tracking accuracy.

To verify the effectiveness of the tracking method described in Section 3.1 and to provide intuitive visualization of the trajectory data in the shape (M,T,P,C), this section presents several examples of point cloud trajectories obtained through our method, as shown in Figure 11. These visualizations directly correspond to the input of the trajectory feature aggregation network (Figure 8), where each subfigure contains up to *M* trajectories (represented by curves of different colors), each trajectory consists of up to T×P points connected in the order of detection over time, and each point carries *C* features (though only spatial coordinates are visualized here).

In Scene 1, a meeting room enclosed by three walls with a high presence of ghosts, one person walks around the environment. The algorithm successfully tracks the trajectory of the real person and also tracks the trajectories of ghosts located behind walls. In Scene 2, where a long wall is present and four people move around the area, the algorithm is able to track all four individuals. Even when two of them pass closely by each other, with their point clouds nearly overlapping, the tracker can still distinguish their respective trajectories.

All of the above trajectory results are obtained using the point cloud pre-segmentation method described in Section 3.2. As a comparison, we also evaluate the tracking results without using pre-segmentation (i.e., with fixed measurement noise covariance). In general, both approaches achieve reasonable tracking performance. However, tracking without pre-segmentation tends to result in more frequent trajectory confusion, especially in narrow environments.

**Figure 12:**
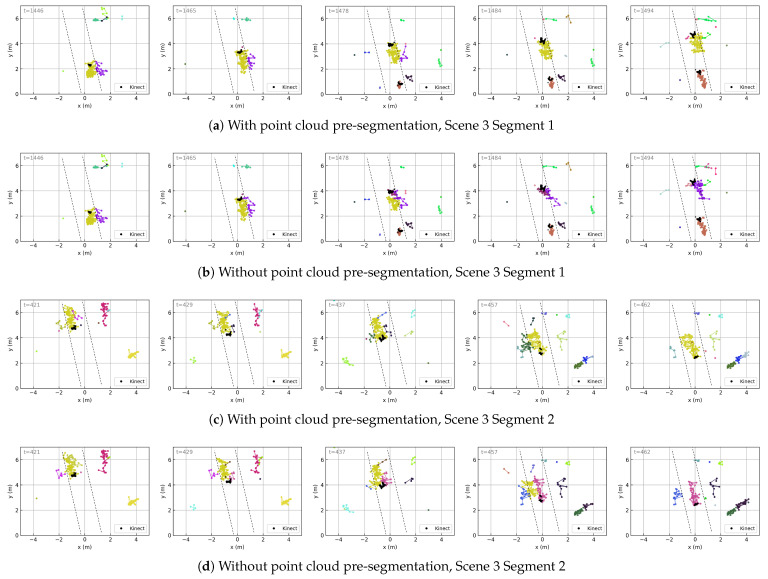
Comparison of trajectories obtained with and without point cloud pre-segmentation. The visualizations follow the same format as in Figure 11.

To highlight the difference, we select a narrow corridor (Scene 3) where ghost points frequently appear near real targets and may capture trajectories, as shown in Figure 12b,d. In contrast, the trajectories generated using point cloud pre-segmentation (Figure 12a,c) demonstrate better stability.

### 4.5. Quantitative Results

The performance of the proposed TrajNet on the test set is summarized in Table 2. It achieves an accuracy of 93.5%, with an AP of 0.992 and an AUROC of 0.982, using only 24.9k parameters. Apart from adopting depthwise-separable convolution, no additional optimization for parameter efficiency has been applied, indicating that the model size could potentially be reduced further.

**Figure 13:**
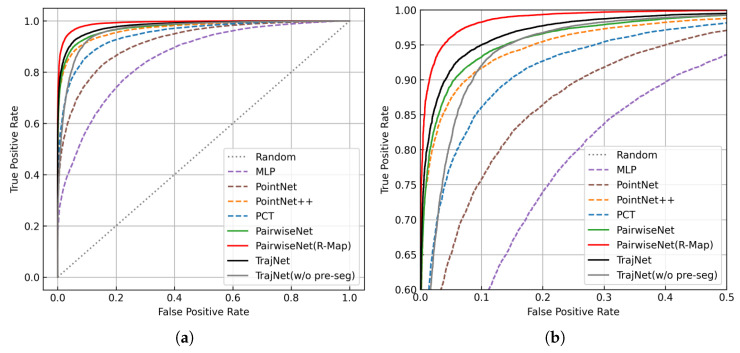
ROC curves of TrajNet and other models. (**a**) Overall ROC curves. (**b**) Zoomed-in view of the ROC curves near the top-left corner.

For comparison, we also report the test performance of several baseline models, including PointNet, DGCNN, PCT, and PairwiseNet, as detailed in [30]. Among all models, TrajNet achieves the best performance among non-map-based methods and is second only to PairwiseNet(R-Map), which leverages environment mapping. This mapping-based approach is less robust in dynamic environments. Once the radar position changes, the map must be regenerated. In contrast, the proposed TrajNet is map-free. Additionally, we plot the ROC curves of all models in Figure 13.

To ensure a fair comparison, we also increase the number of accumulated frames in PairwiseNet from the default 8 to 30, matching the FIFO depth of TrajNet. This variant, denoted as PairwiseNet (T = 30), shows slight performance improvement but still underperforms compared to TrajNet, indicating the latter’s advantage in modeling long-term temporal sequences.

Finally, we also include the performance of TrajNet(w/o pre-seg), which corresponds to the model without point cloud pre-segmentation (i.e., the fourth row in Table 3). Compared to the full TrajNet model, it exhibits a noticeable performance drop, underscoring the importance of pre-segmentation. This observation will be discussed in detail in the next section.

### 4.6. Ablation Study

#### 4.6.1. Component Ablation

To further evaluate the contribution of each component in TrajNet, we conduct a series of ablation experiments, and the results are shown in Table 3. Row 1 represents the default TrajNet model.

Since the point cloud pre-segmentation serves two purposes—assisting tracking by reducing trajectory confusion and enhancing feature representation—we perform ablations for each purpose separately, corresponding to Rows 2 and 3, respectively. Row 4 removes both uses, i.e., completely discards the pre-segmentation.

Row 5 presents the opposite configuration of Row 4—pre-segmentation is retained, but the trajectory-based refinement (i.e., second-stage segmentation) is removed. Based on the first five rows, we conclude that the pre-segmentation and the second-stage segmentation capture complementary information. The main benefit of pre-segmentation arises from the feature-level integration. Based on the architectural differences between the two stages, we speculate that pre-segmentation is better at capturing spatial features, while the trajectory-based refinement (TrajNet stages) excels at leveraging local temporal dynamics, making it more effective in suppressing ghost targets with inconsistent temporal behavior compared to real trajectories.

In addition, we ablate the inter-trajectory feature extraction module described in Section 3.4, as shown in Row 6. A slight performance drop is observed. Furthermore, we replace the pairwise module with an attention mechanism (Row 7), which yields slightly inferior performance compared to the pairwise design.

#### 4.6.2. Input Feature Ablation

We further conduct ablation experiments on the six input features of the original radar point cloud, as summarized in Table 4. Row 1 corresponds to the default TrajNet configuration, where all six features are used.

From the results, we observe that the least important feature is the signal power *P*, followed by the radial velocity *v*. The most critical features are the spatial coordinates *x* and *y*, which contribute more than the range *r* and the angle sinθ.

These results also demonstrate the robustness of the proposed method; even when the input modality undergoes significant changes—for example, when only the *x* and *y* coordinates are retained—the model still achieves reasonably high performance.

### 4.7. Point Cloud Segmentation Results

**Figure 14:**
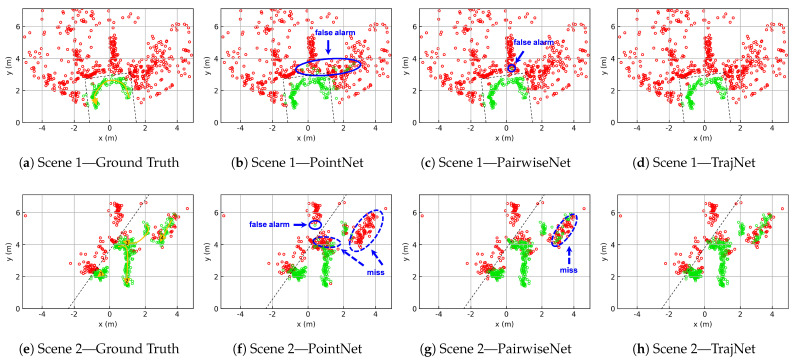
Point cloud segmentation results (green: true targets, red: ghosts). Blue circles highlight key differences among the segmentation outputs of the various networks. Dashed black lines indicate the primary reflective surfaces (i.e., walls). In the ground-truth plots, yellow arrows mark the actual movement trajectories of individuals, and the numbers on the arrows denote their IDs.

To more intuitively demonstrate the effectiveness of ghost suppression, we present the segmentation results of two point cloud sequence segments, as shown in Figure 14. Each sequence spans approximately 5 s and corresponds to Scene 1 and Scene 2 in Figure 11.

In Scene 1, there is only one person, with relatively few real targets and a large number of ghosts. In this scenario, PointNet exhibits multiple false positives and PairwiseNet produces one false positive, while TrajNet achieves completely clean segmentation with no false detections.

Scene 2 is more challenging, involving four individuals. In this case, PointNet suffers from numerous missed detections; both person 3 and person 4 are poorly recognized, and one false positive is also observed. PairwiseNet is able to detect person 3 but struggles to identify person 4. In contrast, TrajNet shows improved recognition of person 4, with only a small number of missed detections.

## 5. Conclusions

This paper proposes a trajectory-based ghost suppression method for mmWave radar. The approach consists of key steps that include point cloud pre-segmentation, inter-frame trajectory tracking, trajectory feature extraction, and broadcasting, effectively integrating spatiotemporal information with point-level features.

The experimental results demonstrate that our method outperforms existing point cloud segmentation approaches, achieving 93.5% accuracy and 98.2% AUROC on the test set. Ablation studies further validate the importance of each component, particularly highlighting that the combination of pre-segmentation and trajectory-based processing captures complementary information that contributes significantly to the overall performance.

Despite efforts to reduce trajectory confusion, the proposed method can still suffer from incorrect associations during tracking. Future work may explore improvements in multi-target tracking robustness. In addition, the current method uses CNN-based temporal feature extraction (chosen for its training stability, parallelizability, and convergence properties) along the trajectory dimension, which requires the explicit storage of a temporal window for each trajectory using a FIFO queue. A promising direction for future research is to replace this design with recurrent neural networks (RNNs), such as assigning a long short-term memory (LSTM) to each trajectory with shared parameters. This could eliminate the need for maintaining historical point storage and reduce redundant computation from sliding windows, enabling more efficient streaming processing.

## Figures and Tables

**Table 1:** Architecture of the temporal CNN using depthwise-separable convolutions.

Stage	Operation	Kernel	Stride	Input Shape	Output Shape
1	Transpose	–	–	(M,30,32)	(M,32,30)
2	DSC 1: Depthwise Conv + ReLU	4	2	(M,32,30)	(M,32,14)
3	DSC 1: Pointwise Conv + ReLU	1	1	(M,32,14)	(M,32,14)
4	DSC 2: Depthwise Conv + ReLU	4	2	(M,32,14)	(M,32,6)
5	DSC 2: Pointwise Conv + ReLU	1	1	(M,32,6)	(M,32,6)
6	DSC 3: Depthwise Conv + ReLU	4	2	(M,32,6)	(M,32,2)
7	DSC 3: Pointwise Conv + ReLU	1	1	(M,32,2)	(M,32,2)
8	Reshape	–	–	(M,32,2)	(M,64)

**Table 2:** Performance comparison between TrajNet and other models.

Model	Acc	Prec.	Recall	F1	AP	AUROC	Params
	(%)	(%)	(%)	(%)	(%)	(%)	
MLP{64,16,2}	80.6	84.5	88.5	86.4	93.1	85.7	1.5k
PointNet [35]	84.5	90.8	86.6	88.7	96.4	91.8	29.7k
PointNet++ [37]	91.3	95.0	92.4	93.7	98.7	97.0	38.7k
DGCNN [38]	91.1	95.1	92.0	93.5	98.6	96.7	227.7k
PCT [39]	88.4	93.9	89.1	91.5	97.8	94.9	629.9k
PairwiseNet [30]	91.8	96.8	91.3	94.0	99.0	97.5	6.2k
PairwiseNet (R-Map) [30]	96.0	96.8	97.5	97.1	99.7	99.2	16.4k
PairwiseNet (T = 30)	92.3	97.0	91.8	94.3	99.1	97.8	6.2k
TrajNet	93.5	95.8	94.9	95.3	99.2	98.2	24.9k
TrajNet (w/o pre-seg)	92.0	95.0	93.5	94.3	98.3	96.4	23.8k

**Table 3:** Performance of TrajNet with different component ablation configurations. A checkmark (✓) indicates that the corresponding component is enabled.

No.	Pre-Seg. for Tracking	Pre-Seg. for Features	Trajectory Aggregation	Pairwise Inter-Trajectory	Attention-Based Inter-Trajectory	Acc	AUROC
1	✓	✓	✓	✓		93.5%	98.2%
2		✓	✓	✓		93.3%	98.1%
3	✓		✓	✓		92.1%	96.5%
4			✓	✓		92.0%	96.4%
5		✓				91.8%	97.5%
6	✓	✓	✓			93.3%	98.1%
7	✓	✓	✓		✓	93.4%	98.1%

**Table 4:** Performance of TrajNet with different combinations of input features. A checkmark (✓) indicates that the corresponding feature is included in the input.

No.	*x*	*y*	*r*	sinθ	*v*	*P*	Acc
1	✓	✓	✓	✓	✓	✓	93.5%
2	✓	✓	✓	✓	✓		93.3%
3	✓	✓	✓	✓		✓	92.7%
4	✓	✓	✓	✓			92.4%
5	✓	✓			✓	✓	90.9%
6	✓	✓					90.3%
7			✓	✓	✓	✓	89.3%

## Data Availability

Due to sponsorship-related restrictions, the dataset is not publicly available but can be shared by the authors upon reasonable request.
